# Influence of visual symptoms on posturographic performance after stroke

**DOI:** 10.1590/2317-1782/20212020262

**Published:** 2023-01-06

**Authors:** Bianca Nunes Pimentel, Valdete Alves Valentins dos Santos

**Affiliations:** 1 Programa de Pós-graduação em Distúrbios da Comunicação Humana - UFSM - Santa Maria (RS), Brasil.; 2 Departamento de Fonoaudiologia, Universidade Federal de Santa Maria - UFSM - Santa Maria (RS), Brasil.

**Keywords:** Postural Balance, Stroke, Vision Disorders, Dizziness, Eye Movements, Equilíbrio Postural, Acidente Vascular Cerebral, Transtornos da Visão, Tontura, Movimentos Oculares

## Abstract

**Purpose:**

to verify the occurrence of visual symptoms in subjects with dizziness after stroke, to compare the posturographic results and to correlate their clinical aspects with the characteristics of the stroke.

**Methods:**

This is an observational, cross-sectional study with quantitative analysis. The inclusion criteria for the sample composition were to report dizziness after ischemic or hemorrhagic stroke and at least 18 years old. We evaluated 50 patients through clinical anamnesis and Dynamic Foam-Laser Posturography. Anteroposterior deviations were calculated with the measures of each SOT. The preferences of the functions were analyzed according to the means of the Sensory Organization Test.

**Results:**

twenty-eight subjects had stroke-related visual symptoms. The prevalent kind of dizziness was imbalance and the most frequent stroke was ischemic, mainly in the carotid territory. The values of tests were below the standard; there was a relationship between older subjects and proprioceptive system, and between visual preference and presence of visual symptoms, as well as the location of the posterior stroke.

**Conclusion:**

there was a high frequency of visual symptoms among subjects with stroke sequelae and these have significant relationship with the worst values in visual preference system.

## RESUMO

**Objetivo:**

verificar a ocorrência de sintomas visuais em sujeitos com tontura após Acidente Vascular Cerebral (AVC), comparar os resultados posturográficos e relacionar seus aspectos clínicos com as características do AVC.

**Método:**

Trata-se de um estudo observacional, transversal, com análise quantitativa. Os critérios de inclusão para composição da amostra foi ter tontura após o AVC isquêmico ou hemorrágico e ter pelo menos 18 anos de idade. Foram avaliados 50 sujeitos por meio de anamnese clínica e os testes da Posturografia Dinâmica Foam Laser. Os desvios anteroposteriores foram calculados de acordo com as médias do Teste de Organização Sensorial.

**Resultados:**

Vinte e oito sujeitos apresentaram sintomas visuais após o AVC. O desequilíbrio foi o tipo de tontura prevalente e o AVC isquêmico o mais comum, sobretudo no território carotídeo. Os resultados dos testes foram inferiores ao padrão de referência; houve relação com sujeitos mais velhos e o sistema proprioceptivo, e entre o sistema de preferência visual e a presença dos sintomas visuais, bem como com o AVC da circulação posterior.

**Conclusão:**

Houve alta frequência de sintomas visuais entre os sujeitos com sequelas de AVC com relação significativa com piores valores da preferência visual.

## INTRODUCTION

Stroke is one of the leading causes of death and disability worldwide, characterized by the acute onset of rapid loss of neurological function, caused by the interruption of blood supply into a particular region of the brain. This type of event causes neurological symptoms such as focal paresis or decrease of motor strength, speech disorders, visual disorders or dizziness, but also global symptoms (coma). Ischemic stroke (IS), which accounts for approximately 80% of cases, occurs when clots block a blood vessel, typically, while a hemorrhagic stroke (HS) results from the rupture of a blood vessel. Consequently, it subsequently generates an accumulation of blood in the brain parenchyma^(1)^.

In Brazil, in the past 30 years, the Brazilian Mortality Information System has reported more than two million deaths resulting from cerebrovascular diseases; 92% of individuals were between 35 to 74 years old^(2)^. From 1990 to 2015, there was a decrease of 46% in mortality. This may represent an increase of survivors living with sequelae; thus, Brazilian authorities need to maintain or even increase investments in prevention, control and treatment of these diseases^(3)^.

It requires greater attention from professionals, especially in the public health scenario, because it affects various aspects of a person’s life. Brain sequelae and health care management, including rehabilitation, are complex to the subjects affected and their families or caregivers. The consequences stemming from that event are diverse, and often sensory-motor deficits affect the maintenance of body balance^(4)^.

Human postural control depends on complex multisensory systems whose information is processed and integrated into the Central Nervous System (CNS)^(5)^. The vestibular system includes structures of the inner ear (labyrinth), which are responsible for sending continuous information about movements and positions of the head and body to integrative centers of the brainstem, to the cerebellum and the somatosensory cortex. The somatosensory system provides proprioceptive input, especially information on joints and axial muscles. The visual system provides sensory inputs from photoreceptors in the retina and visual reflexes by oculomotor systems about the external environment and the position of the body towards such environment^(6)^.

When there are changes in the inputs from this sensory triad, there may occur symptoms such as dizziness, nausea, and feeling of imbalance, among others. Because of this complexity, performance in the maintenance of body balance is vulnerable to illnesses that disrupt any of the systems^(7)^.

Some authors report that patients affected by stroke there may be even higher dependence of eye fixation while maintaining postural balance^(8)^. Different stroke-related visual dysfunctions may occur, depending on the site of the injury, including spontaneous nystagmus^(9)^, changes in eye movements^(10,11)^, and visual perception deficits^(12,13)^, which can affect one or both eyes^(14)^. These changes have a negative impact on the daily life of the affected individuals, and there is little support and provision of information about visual problems of this population^(15)^.

Vestibulometry investigates vestibular function and its relationship with the ocular and proprioceptive systems, cerebellum, spinal cord, reticular formation and nuclei of the brainstem. It is an important diagnostic tool which assists in the recognition of the cause, prognosis and monitoring of the patient’s evolution^(16)^. In outpatient clinics for otoneurological evaluation, stroke is the most frequent etiologic diagnosis among cases of vestibular symptoms arising from impairments in the CNS, and it may reach 40%^(17)^.

Caloric testing is the most commonly used test for vestibular function. The information that composes the evaluation of vectoelectronystagmography is important in patients with visual symptoms and complaint of dizziness after stroke. However, in central pathologies like stroke it is interesting to complement with an evaluation that considers the sensorial integration, especially between the visual preference system and the vestibulospinal reflex. Posturography, in general terms, allows the detection of the deficit sensory system evaluates motor control and assists in the evolution of rehabilitation.

Therefore, the aim of the present study was to verify the occurrence of visual symptoms in subjects with dizziness after stroke, to compare the posturographic results and to correlate their clinical aspects with the characteristics of the stroke.

## METHODS

This is an observational, cross-sectional study with quantitative analysis, registered and approved by the Research and Ethics Committee (REC) under the CPEC (Certificate of Presentation for Ethical Consideration) protocol number 16728013.0.0000.5346.

We selected the subjects from the Audiology Clinic - Department of Speech Therapy - and the University Hospital of a university in shoutern Brazil, between 2015 and 2016. The inclusion criteria for the sample composition were to report dizziness after ischemic or hemorrhagic stroke and at least 18 years old. We exclude the subjects: had a history of visual impairment before the stroke (glaucoma, strabismus, blindness or unilateral or bilateral low vision); vestibular or neurological disorder diagnosed before the stroke; used a wheel chair or depended on a walking stick which prevented the subject from being in a standing position for at least 20 seconds (time of testing the positions in the posturographic exam). Thus, 50 subjects make up the sample. They agreed to participate in the study by reading and signing an Informed Consent Form.

### Procedures

The subjects answered a questionnaire about their clinical history and the stroke (hospitalization, type of injury, sequelae, and rehabilitation), dizziness (onset, characteristics, associated symptoms, diagnosis) and health status in general.

Postural balance was evaluated with Computerized Foam Laser Dynamic Posturography, as proposed by Castagno (1994)^(18)^. Subsequently, we analyze the results of the Sensory Organization Test (SOT) and Sensory Analysis.

The test consists of: a) to be in a standing position (barefoot, bipodal position, arms outstretched along their body inside of a cabinet (1 m^2^ wide and 2 m high, built with demountable iron frames, covered with fabric with horizontal stripes (alternating 10-cm light and dark stripes); b) a belt with a laser pointer was placed around the waist of the subjects, at the level of the 2nd lumbar vertebra. The laser pointer was directed upwards, pointing to a scale on graph paper (50 cm x 50 cm), fixed horizontally on top of the booth by an iron frame. The laser beam was used to assess the anteroposterior displacement of the subjects during the six steps of the SOT, which lasted for 20 seconds each; c) the subjects were informed that there would be a sequence of six separate tests to assess their body sway, with each of them in different situations.

The deviations made by the subjects were recorded on a standard protocol and analyzed on Excel electronic spreadsheet. Anteroposterior deviations were calculated with the measures of each SOT. The preferences of the functions were analyzed according to the means of the SOT tests, according to the following formulas: somatosensory function SOT II/SOT I; visual function: SOT IV/SOT I; vestibular function: SOT V/SOT I; balance index: (SOT III + SOT VI) / (SOT II + SOT IV).

### Statistical analyses

The absolute and relative frequencies were calculated for the categorical variables. Shapiro-Wilk test was used to verify the normality of quantitative variables. To determine the association between the ordinal variables or the continuous variables without a normal distribution, the Mann-Whitney U test and Spearman’s correlation coefficient were used for the correlation, by means of the software STATISTICA 9.1, with a level of significance of 5%, i.e., the results were considered to be statistically significant when *p*< 0.05.

## RESULTS

The sample consisted of 50 subjects, 25 (50%) males and 25 (50%) females with an average age of 63.24 years (±10.61), ranging from 42 to 85 years. All of them had post-stroke complaints of dizziness. Twenty-eight subjects (56%) reported post-stroke visual impairment: blurred vision, reduced acuity, oscillopsia, diplopia and visual field loss. The most prevalent kind of dizziness was imbalance (41-82%) compared with vertigo (nine - 18%); eight subjects (88.89%) had subjective dizziness and one (11.11%), objective dizziness. The most common type of stroke was ischemic (42-84%) compared with hemorrhagic (eight 16%). It occurred in the carotid system in 45 cases (90%) and the vertebrobasilar artery in five cases (10%).

The values of the positions evaluated in the SOT were, on average, below the standard for foam laser posturography (FLP). There was a relationship between older subjects and position II, which evaluates the proprioceptive system (Table 1).

**Table 1:** Average values, medians, minimum and maximum values and standard deviation measured in Foam Laser Dynamic Posturography (FLP) in subjects affected by stroke and the relationship between the results and age (n=50)

Sensory Organization Test (SOT)								
	FLP standard (%)	Mean	Median	Minimum	Maximum	Standard deviation	Age R	Age *p*
SOT I	90	78.81	80.91	34.47	94.27	±10.68	0.01	0.92
SOT II	83	55.03	61.54	-55.81	89.34	±26.86	-0.31	**0.03** *
SOT III	82	43.72	49.70	-54.17	93.63	±29.99	-0.16	0.27
SOT IV	79	54.18	68.46	-44.95	90.14	±37.71	-0.12	0.41
SOT V	60	14.23	20.88	-70.23	84.73	±45.19	-0.12	0.42
SOT VI	54	6.46	10.09	-66.40	68.10	±36.05	-0.08	0.57
Mean		41.29	45.20	-11.75	74.62	±23.70		
Sensory Analysis								
	FLP Pattern %	Mean	Median	Minimum	Maximum	Standard deviation	Age R	Age *p*
SOM	92	69.60	78.50	-71.61	118.92	±33.91	-0.24	0.10
VIS	88	65.92	86.71	-130.39	133.15	±51.94	-0.07	0.64
VEST	67	15.38	27.75	-130.39	109.45	±60.67	-0.11	0.44
PREF	95	5.82	48.45	-541.48	147.36	±152.86	0.01	0.96

Spearman’s correlation test

*p* ≤0.05

Caption: % = Percentage; n = number of subjects; SOM = somatosensory; VIS = visual; VEST = vestibular; PREF = visual preference

* = p <0.05

Women presented higher values in all systems in the sensory analysis, but without significant relation (Table 2).

**Table 2:** Mean values measured in the positions of the Sensory Organization Test (SOT) and Sensory Analysis by sex (n=50)

Sensory Organization Test (SOT)				
	Males (n=25) %	Females (n=25) %	Z	*p*
SOT I	78.16	79.29	0.07	0.94
SOT II	48.60	61.22	1.47	0.14
SOT III	51.38	35.46	-1.45	0.15
SOT IV	46.72	61.81	0.80	0.42
SOT V	7.56	21.23	0.97	0.33
SOT VI	4.56	9.22	0.17	0.86
Sensory Analysis				
SOM	62.03	77.05	1.43	0.15
VIS	55.20	78.11	0.95	0.34
VEST	5.68	25.47	1.10	0.27
PREF	-0.31	10.46	0.04	0.97

Mann-Whitney U Test

*p* ≤0.05

Caption: % = Percentage; n = number of subjects; SOM = somatosensory; VIS = visual; VEST = vestibular; PREF = visual preference; Z = z-value; *p* = p-value

There was no relationship between the classification of stroke and changes in the positions of the SOT (Table 3). However, there was a worse value in position VI among individuals with posterior circulating stroke. (Table 4).

**Table 3:** Distribution and relationship between the values measured in Computerized Foam Laser Dynamic Posturography (FLP) according to type of stroke: ischemic (IS) and hemorrhagic (HS) (n=50)

Sensory Organization Test (SOT)						
	FLP standard (%)	IS (n=42) %	Standard deviation	HS (n=8) %	Standard deviation	*p*
SOT I	90	78.68	±11.31	79.49	±7.03	0.81
SOT II	83	55.30	±27.56	53.56	±24.47	0.53
SOT III	82	43.21	±30.44	46.43	±29.27	0.88
SOT IV	79	55.51	±37.97	47.19	±37.97	0.40
SOT V	60	13.18	±44.29	19.78	±52.56	0.69
SOT VI	54	9.70	±35.96	-10.55	±33.59	0.09
Mean		41.90	±23.93	38.07	±23.73	0.61
Sensory Analysis						
	FLP standard (%)	IS (n=42)	Standard deviation	HS (n=8)	Standard deviation	*p*
SOM	92	70.13	±34.90	66.84	±30.05	0.66
VIS	88	67.51	±53.24	57.53	±46.75	0.34
VEST	67	14.13	±60.51	21.96	±65.26	0.67
PREF	95	15.49	±141.71	-44.97	±206.02	0.08

Mann-Whitney U Test

*p*<0.05

Caption: % = Percentage; n = number of subjects; SOM = somatosensory; VIS = visual; VEST = vestibular; PREF = visual preference; p = p-value

**Table 4:** Distribution and relationship between the values measured in Computerized Foam Laser Dynamic Posturography (FLP) according to affected location by stroke (n=50)

Sensory Organization Test							
Positions	FLP pattern (%)	CA (n=45) %	Standard deviation	VA (n=5) %	Standard deviation	Z	*p*
SOT I	90	78.78	±11.20	79.09	±7.98	0.31	0.76
SOT II	83	58.86	±19.17	46.02	±36.08	0.75	0.45
SOT III	82	43.80	±30.18	42.97	±34.07	0.46	0.65
SOT IV	79	58.86	±34.71	29.19	±48.47	1.74	0.08
SOT V	60	19.70	±41.86	-13.25	±56.10	1.65	0.10
SOT VI	54	10.93	±36.00	-24.99	±21.99	2.26	0.02*
Mean		44.48	±21.81	24.84	±28.27	1.83	0.07
Sensory Analysis							
SOM	92	74.83	±24.44	55.66	±42.35	1.05	0.29
VIS	88	71.84	±49.24	33.85	±61.37	1.95	0.06
VEST	67	22.45	±57.41	-20.75	±69.8	1.68	0.09
PREF	95	17.73	±140.73	-71.39	±231.94	1.92	**<0.05** *

Mann-Whitney U Test

*p*<0.05

Caption: CA = carotid artery; VA = vertebrobasilar artery; SOM = somatosensory; VIS = visual; VEST = vestibular; PREF = visual preference; *p* = p-value

* = p<0.05

There was a relationship between position VI in the SOT and the presence of visual symptoms (Table 5).

**Table 5:** Relationship between presence and absence of visual disorders and values of the Sensory Organization Test (SOT) and Sensory Analysis of the individuals affected by stroke (n=50)

Sensory Organization Test							
Positions	FLP pattern (%)	Without visual complaints (n=22) %	Standard deviation	With visual complaints (n=28) %	Standard deviation	Z	*p*
SOT I	90	80.07	±9.27	77.81	±11.74	0.83	0.40
SOT II	83	54.40	±28.97	55.51	±25.61	-0.05	0.96
SOT III	82	48.32	±22.36	40.11	±34.81	0.67	0.50
SOT IV	79	65.63	±23.79	45.18	±44.16	1.63	0.10
SOT V	60	23.92	±35.61	6.62	±50.83	1.10	0.27
SOT VI	54	19.70	±28.70	-3.95	±38.24	2.12	**0.03** *
Mean		47.75	±18.88	36.21	±26.10	1.56	0.11
Sensory Analysis							
SOM	92	68.68	±38.05	70.32	±30.98	-0.19	0.84
VIS	88	81.37	±27.93	53.77	±62.78	1.48	0.14
VEST	67	29.37	±46.49	4.39	±68.65	1.11	0.26
PREF	95	58.47	±57.12	-35.55	±189.30	2.09	0.04*

Mann-Whitney U Test

*p*<0.05

Caption: % = Percentage; n = number of subjects; SOM = somatosensory; VIS = visual; VEST = vestibular; PREF = visual preference; *p* = p-value

* = p<0.05

## DISCUSSION

Imbalance, a type of non-rotational dizziness, accounts for approximately 15% of all types of dizziness. However, its prevalence is greater in central vestibular syndromes, to the detriment of the occurrence of vertigo. Some of the factors that trigger imbalance are metabolic disturbances, brain injury, and use of specific drugs, in addition to vascular causes^(19)^.

More than half of the sample reported at least one post-stroke visual complaint. Visual disturbances are common in central syndromes, which may lead to changes in visual-vestibular interaction^(19)^.

The mean values measured in the positions in the SOT were lower than the standards of reference^(18)^ and the values in a previous study with stroke patients^(20)^. However, the same authors found a positive influence of the somatosensory system on the balance and functionality of the sample subjects, compared to the other systems evaluated.

An important factor of the present sample is the advanced age of the majority of the subjects. Maintenance of postural balance undergoes physiological and physical changes that are typical of aging. There is a reduction in the speed of inputs, as well as in the processing of responses, which results in greater instability^(21)^. Through posturography, researchers found worse outcomes in the age range of 70-79 years, compared with the age range of 60-69 years, and a decrease of such reduction after 80 years of age^(22)^.

The senescence process alone is not a determining factor in reducing functional capacity, but a set of changes or processes caused by disease and an inadequate lifestyle^(4)^. However, brain plasticity decreases over time^(23)^, so it is more difficult to recover the skills affected by a brain injury. Neuroplasticity is the ability to change the function or the structures of the brain in response to environmental influences. In adult plasticity, according to the morphological manifestation, there is a reduction of the budding of undamaged fibers (axons). In addition, there is no consensus on the processes that involve dendrites and the formation of new synapses^(23)^. Thus, there is clearer evidence that the difference between the performances after an injury is due to reduced ability of older individuals to recover from brain injuries compared with natural aging of systems *per se*.

There was no relation between the positions in the SOT, as well as between sensory analysis, and type of stroke. Hemorrhagic stroke is known to have higher mortality rates than SI, and can lead to more severe sequelae^(24)^. However, an inclusion criterion for this study was the ability to stay in standing position for at least 20 seconds without any kind of help. Consequently, we excluded subjects who were unable to stand, using a cane or wheelchair, many of whom had HS sequelae.

Regarding the relationship between stroke in the posterior circulation and changes in visual preference, it is known that the cranial nerve nuclei involved in visual perception and eye movement control are found in the brainstem. Depending on the affected structures, there will be disturbances of spatial orientation, VOR, posture or neurovegetative manifestations^(6,15,17)^.

The central vestibular pathologies present different clinics according to the etiology, location and extension of the lesion. The presence and characteristics of visual symptoms associated with dizziness, e.g., vertical diplopia, or even the presence and characteristics of nystagmus, are crucial to distinguish peripheral and central syndromes^(25)^.

Signs of central impairment may come from regions directly related to maintaining balance such as cerebellar signs: transverse nystagmus accompanied by gait ataxia and dysarthria; or ocular motility: fixed direction position nystagmus (regardless of the maneuver used), conjugate gaze palsy or vertical nystagmus15 as in pontine infarction. Nevertheless, impairment may occur in other regions, such as thalamic or subthalamic regions, with vertical strabismus, as well as cortical regions, e.g., vascular lesions in the temporal lobe, resulting in paroxysmal seizures, feelings of levitation or falling, visual disturbances due to superior homonymous hemianopsia, secondary to optical radiation injury. These changes may be associated with illusions of displacement and metamorphopsia^(25)^.

In the results of posturography, the group without visual complaints had better performance in five of the six positions, with significant difference in position VI, which represents the visual preference. Visual-vestibular interaction is efficient in the face of spatiotemporal conflicts, such as those evaluated in sensory overload situations. Each sensory system has varying degrees of reliability to realize different parameters of motion. The vision is more sensitive to changes of position and speed, while the vestibular system is more sensitive for detection of acceleration. They also respond differently to conditions of motion: the vision is more sensitive to slow motion while the vestibular system is more sensitive to self-motion fast. Thus, the ideal combination of cues should take into account the reliability of each type of perception^(26)^.

Behavioral and neurophysiological studies show that, when visual and vestibular signals arrive synchronously, they provoke a more reliable behavioral and neuronal response than when presented separately^(27)^. Simultaneous assimilation allows, e.g., discrimination between self-motion and the motion of an object or scene. However, the limits of tolerance of these mechanisms are not yet clear^(28)^.

Patients with visual complaints performed better only in position II of the SOT, evaluated with eyes closed, in which the proprioceptive system prevails. They also showed a higher percentage in sensory analysis in the somatosensory system. In a study with hemiparetic individuals, there was a positive somatosensory influence on balance and functionality of the patients evaluated. This clearly reveals the importance of this component for performance of mobility and functionality in subjects with hemiplegia^(20)^.

When the researchers evaluated posturographic parameters in post-stroke subjects before and after rehabilitation, they found a more significant evolution in the assessment with eyes closed. This difference may be the result of reduced capacity to maintain balance, because of the “temporary loss” of one of the three physiological systems responsible for balance in humans. The fastest activated compensatory mechanism in these situations is proprioception^(29)^.

In position V, the group with visual symptoms had worse values than the group without complaints. This contradicts a previous study in which stroke patients achieved higher values under closed-eye conditions. However, instability during the eyes-open condition does not reduce visual dependence. During recovery, an intervention focusing on eyes-closed exercises can be interesting, because we can train the sensory systems, thus encouraging new control mechanisms (reflexes) to restore support functions and reactions of balance of one lower paretic limb^(30)^.

## CONCLUSION

In this sample of stroke patients, imbalance was the most common type of dizziness. Over half of the subjects reported at least one visual symptom or signs. They showed lower average in five of the six positions evaluated in the SOT, as well as a significant relationship with the worst values in position VI of the SOT and, consequently, in the visual preference system. Decreases in this position are also related to vertebrobasilar stroke, commonly associated with the brainstem and cerebellum, but also supply the occipital lobe responsible for visual processing. The study highlights the importance of the contribution of posturographic evaluation in subjects with sequelae of vascular diseases that cause chronic vestibular-visual symptoms. These evaluations should also be included in the selection of procedures in clinical and therapeutic monitoring.
